# End-tidal carbon monoxide for routine monitoring of significant hemolysis in the management of newborn hyperbilirubinemia

**DOI:** 10.1038/s41372-025-02242-z

**Published:** 2025-02-26

**Authors:** Shanice Wells, Ramya Balasubramanian, Khang Nguyen, David L. Schutzman

**Affiliations:** 1 Department of Pediatrics, Einstein Medical Center Philadelphia, Philadelphia, PA USA; 2 Division of Newborn Medicine, Maria Fareri Children’s Hospital, Westchester Medical Center, Valhalla, NY USA

**Keywords:** Diagnostic markers, Metabolism

## Abstract

**Objective:**

Assess the efficacy of routine ETCOc for all newborns in managing neonatal hyperbilirubinemia.

**Study design:**

Retrospective chart review of 1029 consecutive well-baby nursery admissions following the 2022 AAP hyperbilirubinemia guidelines. Only ETCOc, not type, Rh, and DAT, was used to determine if significant hemolysis was present in sufficient degree to affect bilirubin management. A cost-benefit analysis comparing the two methods was conducted.

**Result:**

2.8% of infants required phototherapy, and 1.1% were readmitted for hyperbilirubinemia. The closer an infant’s bilirubin level was to the phototherapy threshold, the higher the ETCOc. 12 of 29 DAT negative infants with ETCOc ≥ 2.5 PPM who received phototherapy would have gone home with significant hemolysis at risk for readmission or kernicterus if not for the use of ETCOc.

**Conclusion:**

ETCOc is preferable for assessing significant hemolysis in the newborn, can be used to safely manage newborn hyperbilirubinemia, and results in cost savings.

## Introduction

Hyperbilirubinemia is a common condition in newborns [1]. Hemolysis has been recognized as a risk factor for severe hyperbilirubinemia since at least 1932 [2], and algorithms adjusting phototherapy levels based on the presence of hemolysis have been in use since at least 1970 [3]. The American Academy of Pediatrics (AAP) has recognized significant hemolysis to be a risk factor for bilirubin induced neurological damage, and has provided specific recommendations for lowering the threshold for initiating phototherapy if significant hemolysis is present starting in 2004 [4].

End tidal carbon monoxide corrected for ambient carbon monoxide (ETCOc) has been recommended as a method to determine degree of hemolysis since 2004 [4]. Until recently, however, that method was not easily performed in a clinical setting. In its place, direct antibody test (DAT) screening has been widely used to assess the presence of hemolysis, primarily due to its universal availability and rapid turn-around time. However, multiple studies have shown that DAT+ is often not associated with significant hemolysis and did not predict the severity of neonatal hyperbilirubinemia [5–7]. ETCOc is a much more specific measure of hemolysis, and it can now be easily and non-invasively measured via Capnia’s CoSense ETCO monitor (CoSense, Capnia, Redwood City, CA, USA) on an infant’s exhaled breath.

Our unit first began using ETCOc to determine hemolysis as part of a large, multi-center study evaluating the Capnia CoSense monitor [8]. Following that study, we continued to test infants who were either DAT+ or in the high-intermediate risk or high-risk zone of the Bhutani nomogram [9]. With the initiation of the 2022 AAP guidelines [10], and having seen that significant hemolysis was usually not present in supposedly high risk infants [9], we decided to evaluate a cohort of consecutive admissions to the well-baby nursery for the presence of significant hemolysis using ETCOc and compare their need for phototherapy and readmission with the common risk factor of DAT + .

## Subjects and methods

Data was collected from 1094 consecutive admissions to the well-baby nursery of Einstein Medical Center Philadelphia from 8/9/2023 to 3/21/2024. Based on standard unit protocols, blood type, Rh, and DAT testing was performed on cord blood collected on all babies whose mothers were either type O or Rh negative. A CBC and reticulocyte count was performed on all infants with A/B/O incompatibility. An ETCOc level was collected on each infant, usually in the mother’s post-partum room, at the time of the infant’s hearing screening. The device used was the Capnia CoSense monitor. The device was approved by the FDA in 2014, and multiple studies since have shown it to meet the standards required to detect hemolysis in infants [11]. Although the device might not be able to obtain a level at times due to a rapid respiratory rate or elevated H^+^ level [12], ultimately, we were able to obtain an ETCOc level on all infants in the study. An ETCOc level ≥ 2.5 PPM was considered evidence of significant hemolysis, as this was the 95% for infants from our unit in a previous multi-center study [8]. In the current study, criteria for bilirubin management were based on the AAP guidelines in place at the time of the infants’ birth [10], and an ETCOc level ≥ 2.5 PPM was used as the criterion for significant hemolysis as opposed to DAT + . Follow-up for hyperbilirubinemia after discharge was based on the findings of Kuzniewicz et al. [13]. Infants who required admission to the neonatal intensive care unit either before or after admission to the well-baby nursery, except only for the provision of phototherapy, were removed, leaving a total of 1029 infants to be part of the study.

Demographic data was collected, and an analysis of the need for phototherapy using either DAT or ETCOc as the criterion for hemolysis was conducted. A cost-benefit analysis comparing the two methods was also performed. This study was approved by the institutional review board (Approval # 2023-1055).

## Results

**Fig. 1:**
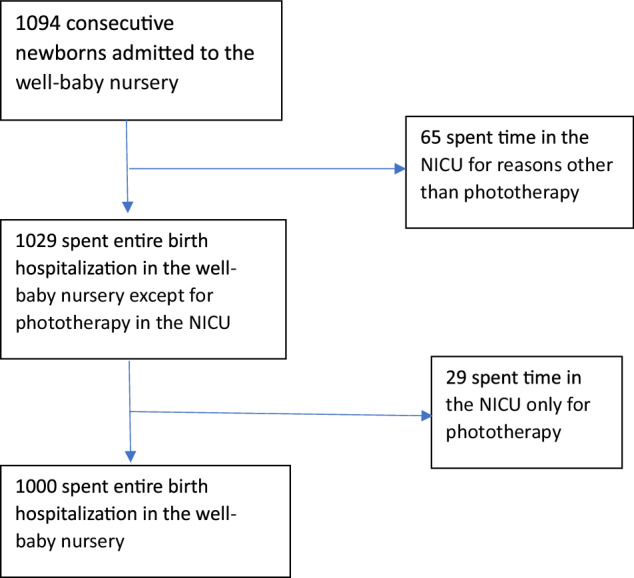
Flow diagram of patient enrollment.

The demographics of the study population are noted in Table 1. Of the 1094 infants initially admitted to the nursery, 65 were removed from the final results as they had spent a portion of their birth hospitalization in the neonatal intensive care unit (NICU) for various reasons. Of the remaining 1029 infants, 29 underwent phototherapy in the NICU, had no other indications for NICU admission, and are the subject of this report. A total of 1000 infants remained in the well-baby nursery for the entirety of their birth hospitalization (Fig. 1). Of the 1029 infants studied, 29 (2.8%) required phototherapy during their initial birth hospitalization, and 11 of 1029 (1.1%) required readmission for phototherapy after their initial discharge. Only one infant who required phototherapy during their birth hospitalization was subsequently readmitted for phototherapy. The breakdown of the group by sex, gestational age, and race or ethnicity is presented in Table 1. A total of 30 (2.9%) infants were DAT positive. A total of 48 (4.7%) were G6PD positive based on their state mandated newborn screen. Since the newborn screen results were generally not available until approximately one week after birth, the G6PD results were not used as a criterion for significant hemolysis. The average age at ETCOc collection was 32.5 ± 8.7 h (Table 2). Table 2 also presents ETCOc levels stratified by the need for phototherapy or readmission, race or ethnicity, mg/dL below phototherapy of the discharge bilirubin, and relationship of DAT positive to ETCOc. A cost/benefit analysis is presented in Table 3. A total of 7 fewer infants received phototherapy using ETCOc as opposed to DAT+ as a marker of significant hemolysis, and 5 fewer infants were readmitted for phototherapy by using ETCOc instead of DAT + . The costs for laboratory studies were provided by the hospital’s lab supervisor, and the room and board costs were provided by the hospital’s financial department. We assumed a 2-day increased length of stay for either phototherapy during the initial birth hospitalization or readmission. We also assumed two serum bilirubin tests for each additional day. The mothers of 447 infants were O+ and 116 infants had A/B/O incompatibility with their mothers. The analysis revealed a savings of $13,123.00 for the entire group by using ETCOc instead of DAT+ to determine significant hemolysis.

**Table 1:** Demographics.

	Exclusive well-baby nursery	Phototherapy	Readmissions
*N*	1029	29 (2.8%)	11 (1.1%)
Male	522 (50.7%)	16 (55.2%)	6 (54.5%)
Female	507 (49.3%)	13 (44.8%)	5 (45,5%)
Gestational Age (wk)			
35	7 (0.7%)	1 (3.4%)	1 (9.1%)
36	45 (4.4%)	3 (10.4%)	1 (9.1%)
37	129 (12.5%)	4 (13.8%)	3 (27.3%)
38	216 (21.0%)	3 (10.4%)	3 (27.3%)
39	432 (42.0%)	11 (37.9%)	3 (27.3%)
40	145 (14.1%)	7 (24.1%)	0 (0.0%)
41	55 (5.3%)	0 (0.0%)	0 (0.0%)
Ethnicity*			
AA	483 (46.9%)	15 (51.7%)	6 (54.5%)
H	353 (34.3%)	9 (31.0%)	2 (18.2%)
C	62 (6.0%)	0 (0.0%)	0 (0.0%)
A	50 (4.9%)	3 (10.4%)	2 (18.2%)
O	81 (7.9%)	2 (6.9%)	1 (9.1%)
ABO incompatible			
Baby A	87 (8.5%)	7 (24.1%)	1 (9.1%)
Baby B	44 (4.3%)	8 (27.6%)	2 (18.2%)
Baby A DAT pos	14 (1.4%)	4 (13.8%)	0 (0.0%)
Baby B DAT pos	14 (1.4%)	4 (13.8%)	0 (0.0%)
Baby O DAT pos	2 (0.2%)	0 (0.0%)	0 (0.0%)
G6PD pos	48 (4.7%)	3 (10.3%)	4 (36.4%)

*AA African American, *H* Hispanic, *C* Caucasian, *A* Asian, *O* Other

**Table 2:** ETCOc levels.

**A**	**ETCOc mean (ppm)**	
All infants	(*n* = 1029)	1.7 ± 0.5
Phototherapy	(*n* = 29)	2.7 ± 0.7
Readmission	(*n* = 11)	2.2 ± 0.4
**B**	**Average age at ETCOc (hr)**	
All infants	32.5 ± 8.7	
Phototherapy	33.1 ± 13.7	
Readmission	36.0 ± 7.6	
**C**	**ETCOc by ethnicity (ppm)**	
African American	1.7 ± 0.5	
Hispanic	1.8 ± 0.5	
Caucasian	1.8 ± 0.5	
Asian	1.7 ± 0.5	
Other	1.7 ± 0.5	
**D**	**ETCOc (ppm) by mg/dL (Δ) below phototherapy level**	
Δ < 3 (*n* = 21)	2.5 ± 0.4	
Δ 3–5 (*n* = 118)	2.0 ± 0.5	
Δ ≥ 5 (*n* = 861)	1.7 ± 0.4	
**E**	**ETCOc and DAT**	
30 of 1029 DAT+ (2.9%)		
7 of 30 (23.3%) DAT+ with ETCOc ≥ 2.5 ppm		
84 of 999 (8.4%) DAT neg with ETCOc ≥ 2.5 ppm

**Table 3:** Cost/benefit analysis.

Savings	
Cost of Rh and Type	$15,609
($34.92/test x 447 tests)	
Cost of DAT	$6777
($15.16/test x 447 tests)	
Cost of CBC	$686
($5.91 per test x 116 tests)	
Cost of reticulocyte count	$601
($5.18/test x 116 tests)	
Cost of lancet for bilirubin test	$139
($2.90 per lancet x 4 for each 2-day stay)	
Cost of tubes for bilirubin test	$25
($0.59 per tube x 4 for each 2-day stay)	
Cost to perform each bilirubin test	$420
($8.75 per test x 4 for each 2-day stay)	
NICU room and board	$36,126
(12 additional patients each with 2-day LOS and $1509 cost per day)	
Saving per 1029 infants	$54,283
Cost of cannulas ($40.00/cannula)	$41,160
NET savings per 1029 infants	$13,123

## Discussion

Recently, Christensen et al. [11] published a paper describing ETCOc as the gold standard for determining hemolysis in newborns. Multiple studies have found that many DAT+ infants are in fact not hemolyzing when assessed by either ETCOc or carboxyhemoglobin levels [5–7]. A total of 30 (2.9%) infants in this study were DAT+ (Table 2E), and only 7 (23.3%) showed evidence of significant hemolysis. This incidence of significant hemolysis is similar to prior cohorts from our institution when either ETCOc [7] or COHbg [6] were used as a measure of hemolysis. More concerning is the fact that 84/999 (8.4%) infants in this study who were either DAT negative, or did not have their DAT evaluated, in fact had significant hemolysis with ETCOc levels ≥2.5 PPM (Table 2E).

ETCOc is a simple, non-invasive test that can be used to safely manage newborn hyperbilirubinemia with no additional staff time required. In this study, ETCOc levels were measured by unit staff during performance of the hearing screen. While waiting for the hearing screening device to complete a test, there was plenty of time to determine the infant’s ETCOc level. ETCOc levels have been shown to be relatively stable during the first three days of an infant’s life [14], so each post-partum unit can determine how best to fit the test into their routine.

In this study, using the 2022 AAP guidelines and ETCOc as a measure of hemolysis, 2.8% of the infants required phototherapy, and only 1.1% of the cohort were readmitted. This is substantially better than the levels found by Cahill, et al. [15] when they compared their incidence of phototherapy and readmission using the 2004 and 2022 AAP guidelines, and similar to the results of Sarathy et al. [16] who also used the 2022 guidelines. ETCOc levels were identical between those in our entire group and those who were neither readmitted nor underwent phototherapy (1.7 ± 0.5 PPM vs. 1.7 ± 0.5 PPM, respectively). It was higher for those infants requiring phototherapy as compared to those who did not, and, not surprisingly, the closer an infant’s bilirubin level was to their phototherapy level, the higher their ETCOc (Table 2A and 2D, respectively). Infants who were readmitted had ETCOc levels intermediate between those who required phototherapy and the cohort of all infants (Table 2A).

The ETCO levels among the different races and ethnicities in our cohort were quite similar ranging from 1.7 ± 0.5–1.8 ± 0.5 PPM, with 2 standard deviations above the mean ranging from 2.7–2.8 PPM. Different mean ETCOc levels have been found among various cohorts of infants. In a group of Chinese infants [14], 95% was found to be 2.2 PPM. Christensen et al. [17] found the 95% to be 1.7 PPM in a primarily Caucasian population. Similarly, there are various recommendations regarding what level to use as significant hemolysis. While we used 2.5 PPM, Bilitool (Bilitool.org) uses a cutoff of 1.7 PPM which is the 75% noted by Bao et al. [14]. Since each site will have a population with somewhat different ETCOc values, a trial period to determine mean ETCOc levels, and at which percentile the unit feels comfortable determining significant hemolysis, should be implemented before beginning to use ETCOc as the marker of hemolysis.

In 2021 Kuzniewicz et al. [13] reviewed close to 150,000 infants born in the Northern California Kaiser Permanente system. They determined how many mg/dl their discharge bilirubin was below the appropriate phototherapy level and then determined the rate of readmission for each 1 mg cohort. They recommended 24-h follow up for a discharge bilirubin 0–2 mg/dL below the phototherapy level, follow-up in 48 h for a bilirubin level 2–3 mg below the phototherapy level, and clinical follow-up alone for a discharge bilirubin more than 3 mg/dL below the phototherapy level. They reviewed their data again after publication of the 2022 AAP guidelines and found their recommendations still held [18]. In order to be conservative, infants in this study had routine follow-up at 24 h after discharge for a bilirubin less than 3 mg/dL below phototherapy levels, 48-h follow-up for a bilirubin 3–5 mg/dL below phototherapy level, and clinical follow-up alone for a bilirubin more than 5 mg/dL below phototherapy levels. Of the 1000 infants who did not require phototherapy, 21 (2.1%) had a discharge bilirubin <3 mg/dL below the phototherapy level, and 118 (11.8%) had a discharge bilirubin between 3 and 5 mg/dL below their phototherapy level. Thus only 13.9% of our infants required follow-up after discharge which is similar to Kuzniewicz et al. [13] and substantially fewer than the 60% of infants requiring some reevaluation using the Bhutani nomogram [9]. Unsurprisingly, ETCOc levels were higher the closer the discharge bilirubin level was to the phototherapy level (Table 2D). Using their nomogram, Kuzniewicz et al. [13] had a readmission rate of 0.3% for infants whose discharge bilirubin was more than 5.5 mg/dL below phototherapy level. In our study, 2 of 1029 infants were readmitted with a discharge bilirubin >5 mg/dL below phototherapy (0.2%) which is comparable to the results of Kuzniewicz et al. [13].

As noted above (Table 3), by using ETCOc instead of DAT+ to determine the presence of significant hemolysis, a total of $13 123.00 could be saved for this group of 1029 newborns. The major source of savings was the ability to eliminate the type, RH, and DAT on infants whose mothers were type O+ and the room and board savings for the 12 fewer infants who would either not require phototherapy or would not be readmitted. Another source of savings, or more properly a lack of increased cost, occurred by performing ETCOc at the time of the newborn’s hearing screen. Using this method resulted in no additional time spent by any hospital staff to obtain the results. Potentially the most important source of savings is decreasing the risk of sending infants home with significant hemolysis who were not identified. In this study, 12/29 (41.4%) of the infants requiring phototherapy were either DAT negative or did not have their DAT measured. A total of 84 of the 999 (8.4%) infants in the entire group who were either DAT negative or did not have their DAT measured had significant hemolysis (ETCOc ≥2.5 PPM). These infants have an increased risk of significant hyperbilirubinemia, and potentially kernicterus, that might not be picked up until significant neurological damage has occurred. Unfortunately, the current legal system in the United States does not function well to accurately determine true medical malpractice. One of the authors (DLS) has served as a defense expert in 2 malpractice cases where there was no evidence of malpractice [19, 20]. However, the plaintiffs presented the two children with significant neurological deficits during their trials. In one case [19], the jury awarded the plaintiff a judgment in excess of $100,000,000, and in the other case [20], the parties reached a multimillion-dollar settlement. Since the awards in kernicterus cases run between 10 and 50 million dollars [21], avoiding one case of kernicterus would pay for nasal cannulas to perform ETCOcs on the newborns in our institution for 125 years.

In summary, ETCOc is a simple, non-invasive test that is the current gold standard for determining hemolysis in a newborn [11]. By using ETCOc instead of DAT+ to determine hemolysis, fewer infants will require phototherapy during their birth hospitalization, and fewer infants will be readmitted to the hospital for phototherapy after their initial discharge. In addition to the health benefits to these infants and families, there would be monetary savings to the hospitals, and potentially the avoidance of cases of kernicterus and all of its disastrous consequences.

## Data Availability

Data can be obtained on appropriate request from the corresponding author.
